# Comparing individually tailored to disorder-specific internet-based cognitive–behavioural therapy: benchmarking study

**DOI:** 10.1192/bjo.2018.41

**Published:** 2018-07-18

**Authors:** Martin Kraepelien, Erik Forsell, Eyal Karin, Robert Johansson, Nils Lindefors, Viktor Kaldo

**Affiliations:** Department of Clinical Neuroscience, Centre for Psychiatry Research, Karolinska Institutet, Sweden; Department of Clinical Neuroscience, Centre for Psychiatry Research, Karolinska Institutet, Sweden; eCentreClinic, Department of Psychology, Macquarie University, Australia; Department of Clinical Neuroscience, Centre for Psychiatry Research, Karolinska Institutet and Department of Psychology, Stockholm University, Sweden; Department of Clinical Neuroscience, Centre for Psychiatry Research, Karolinska Institutet, Sweden; Department of Clinical Neuroscience, Centre for Psychiatry Research, Karolinska Institutet and Department of Psychology, Faculty of Health and Life Sciences, Linnaeus University, Sweden

**Keywords:** cognitive–behavioural therapies, comorbidity, depressive disorders

## Abstract

Disorder-specific internet-based cognitive–behavioural therapy (ICBT) is effective for depression, panic disorder and social anxiety. In this benchmarking study, a new, individually tailored, ICBT programme (TAIL) showed effects on depression (*n* = 284, *d* = 1.33) that were non-inferior to disorder-specific ICBT for depression in routine care (*n* = 2358, *d* = 1.35). However, the hypotheses that TAIL for individuals with social anxiety or panic disorder is inferior to disorder-specific ICBT could not be rejected (social anxiety: TAIL *d* = 0.74 versus disorder-specific *d* = 0.81; panic: TAIL *d* = 1.11 versus disorder-specific *d* = 1.47). Our findings strengthen the empirical base for TAIL as an alternative to disorder-specific ICBT for depression.

**Declaration of interest:**

None.

Disorder-specific, therapist-guided internet-based cognitive–behavioural therapy (ICBT) is established as efficacious and cost-effective for depression, panic disorder and social anxiety.^1^ It has been implemented in routine care in Sweden since 2007.^2^^–^^4^ However, depression and anxiety disorders are often comorbid.^5^

Individually tailored ICBT^6^ handles comorbidities within one treatment by prescribing different cognitive–behavioural therapy components matched to the individual's needs. This can manage comorbid conditions more optimally and lessen the need for thorough diagnostic procedures and undertaking several disorder-specific treatments.

One trial^6^ indicated no difference in reduction of depressive symptoms between individually tailored ICBT and disorder-specific ICBT. In that study, individually tailored treatment was even more effective than disorder-specific treatment in reducing depression in a subgroup with higher levels of depression and comorbidity. However, larger comparison groups are needed to explore the non-inferiority of individually tailored to disorder-specific ICBT. Also, the effects of individually tailored ICBT for depression on comorbid conditions have not been explored.

Our primary aim was to use the basic methodology of benchmarking to compare a new intervention with a more established one. We evaluate if the effects of an individually tailored ICBT programme (TAIL) on depressive symptoms among patients in TAIL with depression (TAIL-depression) were non-inferior to the effects of a disorder-specific ICBT for depression used in routine care (DS-depression). Secondary aims were to use the same benchmarking strategy to compare reduction in anxiety among patients in TAIL with either panic disorder (TAIL-panic) or social anxiety disorder (TAIL-social anxiety) with disorder-specific ICBT in routine care for patients with panic (DS-panic) or social anxiety (DS-social anxiety).

## Method

The treatment samples that served as the benchmarks of this study were clinical outcomes from disorder-specific ICBT within a routine care service at the internet psychiatry clinic^2^^–^^4^ in Stockholm, Sweden from October 2007 to July 2017. Comorbid anxiety and depression in this sample, receiving disorder-specific ICBT for panic disorder, depression and social anxiety disorder, was 14, 25 and 15% respectively. The alternative, comparator treatment sample, was collated from individually tailored ICBT given at the same clinic, but within the context of a trial.^7^ Comorbid anxiety and depression was 67% in this sample. Details on the sample, trial methodology and clinical effects are found in previously reported results from the REGASSA study.^7^ In that study TAIL was superior to treatment as usual in primary care in reducing depression. Details on ethical registration, treatment content, inclusion criteria, diagnostic procedures and the study flow chart of the current study can be found in the supplementary Data 1 and supplementary Fig. 1 available at https://doi.org/10.1192/bjo.2018.41.

The measures, employed at pre-treatment and post-treatment 12 weeks later in both treatment samples, include well-established disorder-specific self-report scales: the Montgomery–Åsberg Depression Rating Scale – Self rated (MADRS-S),^8^ the Panic Disorder Severity Scale – Self report (PDSS-SR)^9^ and the Liebowitz Social Anxiety Scale – Self-Rated (LSAS-SR).^10^ Measurements were collected weekly in the disorder-specific treatment. In TAIL only MADRS-S was collected weekly whereas PDSS-SR and LSAS-SR were collected before and after treatment. Demographics and data on treatment use was collected from both TAIL and disorder-specific ICBT. Self-rated data from TAIL has not been previously published.

Clinical change within each group was estimated through longitudinal generalised estimating equations models, with each of the symptom outcomes modelled separately. These models tested for both differences in the symptom scores between groups at baseline, as well as for group differences in the rate of symptom reduction (group × time interaction) as an additional exploratory analysis.

In addition, in line with methodological recommendations for clinical measurement and reporting,^11^ demographical variables such as age, employment and relationship status variables were used to adjust the longitudinal models, and the test between groups. Specifically, those variables that illustrated a significant statistical association to either the overall symptom score, or the rate of improvement, were retained in the models that test for differences between groups. Additional information about the confounding variables, adjusted and unadjusted estimates of change is presented in supplementary data 1.

The estimated marginal means from the resulting longitudinal models were also used to determine within-group effect sizes (Cohen's *d*) and percentage score estimates that conveyed the clinical change through both treatment conditions. In addition, the frequency of individuals who demonstrated a clinical response, defined as a 50% reduction in symptoms, was tested for between groups through a logistic regression.

The main analysis of this paper employed a benchmarking strategy proposed by Minami and colleagues.^12^ This procedure does not rely on the traditional statistical tests for differences described above. Instead, the within-group effect size of the treatment would be considered non-inferior, or ‘clinically equivalent’ using Minami and colleagues’ terminology, to the effect size of the benchmark treatment if the lower end of the 95% CI round the treatment mean effect size is at least as large as the benchmark mean effect size minus the minimally clinically relevant effect (Δ). This so called ‘good enough principle’, originally proposed by Serlin & Lapsey,^13^ requires a value of Δ to be chosen to reflect the best available knowledge in the field. We chose to use Δ = 0.24 as the minimal clinically relevant effect following the empirically derived proposal for depression treatment by Cuijpers and colleagues.^14^ Thus, if the effect of disorder-specific treatment minus 0.24, is lower than the lower end of the 95% CI of the effect of the individually tailored treatment, the treatment is considered non-inferior to the benchmark.

## Results

Demographical information and information about use of treatment is available in supplementary Table 1. Comparisons between the main groups of TAIL and disorder-specific treatment for depression demonstrated slight differences of age, employment rate and sent messages with no other significant differences between the groups.

Table 1 reports estimated symptom levels and statistical tests. Initial severity scores did not differ significantly (*P* = 0.28–0.68 as described in the similarities and differences section in the supplementary material). Supplementary Table 2 reports the adjusted estimates. Since there were no changes to the unadjusted test results, the unadjusted model is used as the main analysis.

**Table 1:** Comparison of disorder-specific and individually tailored treatments

	Estimated marginal means					Benchmarking parameters, percentage change and response rates					
Condition (scale) and group	*n*	Pre-treatment (s.d.)	Post-treatment (s.d.)	G × T, W*χ*^2^	G × T, *P*	Effect size, *d* (95% CI) (within group)	*d* – Δ	Percentage change (95% CI)	>50% response (95% CI)	Response, W*χ*^2^	Response, *P*
Depression (MADRS-S)											
DS-depression	2358	23.57 (5.76)	13.81 (8.43)	0.00		1.35 (1.29–1.42)		41.3 (39.7–42.8)	43.5 (41.4–45.7)	0.51	
TAIL-depression	284	22.98 (5.90)	13.50 (8.19)		0.98	1.33 (1.14–1.52)	1.11	41.3 (36.7–45.6)	45.9 (39.8–52.2)		0.47
Panic (PDSS-SR)											
DS-panic	1176	12.30 (4.30)	5.56 (4.77)	3.52		1.47 (1.37–1.56)		54.5 (52.0–56.9)	61.9 (58.8–64.9)	1.02	
TAIL-panic	54	12.02 (4.46)	6.67 (5.20)		0.06	1.11 (0.66–1.56)	1.23	43.9 (29.0–55.6)	53.8 (38.3–68.6)		0.31
Social anxiety (LSAS-SR)											
DS-social anxiety	1335	73.15 (22.67)	53.79 (25.35)	0.22		0.81 (0.73–0.90)		26.5 (24.5–28.5)	18.3 (16.2–20.7)	0.01	
TAIL-social anxiety	46	70.50 (23.25)	53.45 (23.66)		0.64	0.74 (0.27–1.21)	0.57	24.2 (12.8–34.1)	17.6 (8.1–34.1)		0.92

DS, disorder-specific treatment; TAIL, individually tailored treatment; G × T, interaction effect of group and time from generalised estimating equations; W*χ*^2^, Wald chi-squared; *d*, Cohen's *d*; *d* – Δ, non-inferiority margin; MADRS-S, Montgomery–Åsberg Depression Rating Scale – Self-rated; PDSS-SR, Panic Disorder Severity Scale – Self report; LSAS-SR, Liebowitz Social Anxiety Scale – Self-Rated.

For the TAIL-depression group, the within-group effect size of *d* = 1.33 (95% CI 1.14–1.52) was above the non-inferiority margin of the DS-depression group (*d* = 1.35, *d* − Δ = 1.11). The effect size of the TAIL-panic group *d* = 1.11 (95% CI 0.66–1.56) was slightly lower than the effect of the DS-panic group and not above the non-inferiority margin (*d* = 1.47, *d* − Δ = 1.23). The effect size of the TAIL-social anxiety group was 0.74 (95% CI 0.27–1.21) and similar to the effect of the DS-social anxiety group, but the lower end of the confidence interval was not above the non-inferiority margin (*d* = 0.81, *d* − Δ = 0.57).

In the additional testing for differences in symptom reduction, no TAIL intervention was significantly different from any of the disorder-specific treatments, although TAIL-panic showed a close to significantly lower decrease of panic symptoms.

## Discussion

When benchmarking individually tailored ICBT to well-established disorder-specific ICBT programmes in routine care, we demonstrated that the effect of tailored treatment for patients with depression was non-inferior to disorder-specific treatment on depression. The effect on comorbid panic was at least of moderate size but non-inferiority could not be established. In addition, the effect size point estimate was 0.36 lower than the effect of the disorder-specific panic treatment, and the exploratory test for difference between TAIL-panic and DS-panic was near statistical significance (*P* = 0.06). The effects on comorbid social anxiety had a similar point estimate as the effects of disorder-specific social anxiety treatment but non-inferiority could not be established.

One limitation was the low power in the benchmark analyses for participants with panic or social anxiety, calling for future studies with larger samples. Also, some minor differences in sociodemographic factors and interaction during treatment were found, and the diagnostic procedures and the context (randomised controlled trial versus routine care) differed. However, it is not obvious which treatment would benefit overall from these differences and many important conditions were very similar, for example the use of the same technical platform, length of treatment, level and mode of support, therapist's competence and initial severity. The result was also robust to controlling for demographical variables. Another limitation was that the benchmarks were routine care treatments, whereas the context of the comparator intervention was that of a randomised controlled trial. However, these disorder-specific treatments were run in a context not very different from a clinical trial and in many ways similar to how the REGASSA study was carried out (see the similarities and differences-section in the supplementary material for comparison).

Individually tailored ICBT for patients with depression was identified as non-inferior to disorder-specific ICBT for depression. For the subgroup of patients with social anxiety disorder, the reductions in anxiety were comparable with those found in disorder-specific ICBT for social anxiety, although the low statistical power does not admit conclusions about non-inferiority. For panic disorder the disorder-specific ICBT reduces anxiety almost significantly more than individually tailored ICBT, and the difference in effect size point estimates is greater than the non-inferiority margin, giving a non-significant but suggestive notion that there could be a clinically relevant advantage of selecting disorder-specific ICBT when the patient has panic disorder. Therapist-time was also significantly greater in TAIL for patients with social anxiety and panic disorder, which suggests lower cost-effectiveness with regards to the effects on the patient's main problem.

The empirical findings in this study do not provide any suggestions on why depression might be more suitable to treat with individually tailored ICBT than anxiety, but in theory it is in line with the finding that a wide range of interventions seems to be equally effective (or ineffective) for depression,^15^ indicating less need for specific treatment components, whereas this might not be the case for anxiety where, for example, exposure seems to be a technique of specific importance. If this is the case, and if tailored treatments might not focus enough on exposure exercises, this could be one of several explanations for our observed difference.

In conclusion, although more, highly powered, research on individually tailored ICBT for social anxiety and panic is needed, our findings support the use of individually tailored ICBT as an alternative for patients presenting with depression as the primary condition of concern. The increased use of individually tailored ICBT, where a patient is allowed to have more than one key treatment target, could lead to less need for highly precise diagnostic procedures or having to undertake several disorder-specific treatments.

## Supplementary material

For supplementary material accompanying this paper visit http://dx.doi.org/10.1192/bjo.2018.41. click here to view supplementary material
