# Supplementary material for: Comparing individually tailored to disorder-specific internet-based cognitive–behavioural therapy: benchmarking study
Source: BJPsych Open. 2018 Jul 18;4(4):282–4. doi: 10.1192/bjo.2018.41 (PMC6066990; doi:10.1192/bjo.2018.41)
Supplement: Supplementary file 1 [file S2056472418000418sup001.docx]

**Supplementary Data 1**

**Trial registration and ethical approval**

The REGASSA study was registered with the registry of clinical trials in Stockholm county (KCTR study ID: KT20110063). The REGASSA study and the disorder-specific treatment-studies were approved by the Regional Ethics Review Board in Stockholm, Sweden (2010/1779-31/4, 2011/2091-31/3).

**Treatment content of TAIL**

The treatment content was based on CBT, and consisted of text-based modules that the participants worked with for a week per module, on average. They ended with a homework assignment that the patient would send to the therapist. The therapist would then give feedback and unlock the next module. The first three modules were given to all participants and were focused on depressive symptoms, inactivity and avoidance behaviors. During these first three weeks, the participant filled out online questionnaires about different symptoms and received a telephone call from their therapist in order to guide the selection of suitable treatment modules. From week four, the treatment content was tailored to the individual participant and could include, for example, psychoeducation about panic or social anxiety and graded exposure to overcome anxiety and avoidance behaviors. There was also tailored content about worry, stress, insomnia and pain. The last module summarized treatment and included relapse prevention. Participants could always write to their therapist and receive an answer within two days. If a patient had been inactive for a week, they were prompted to log in by text message or a short phone call and therapists always gave thorough feedback on the homework reports patients submitted at the end of each module.

**Similarities and differences between the TAIL and DS treatments and contexts**

TAIL was given on the same treatment platform as the disorder-specific treatments i.e. [www.internepsykiatri.se](http://www.internepsykiatri.se), a secure website operated by the Stockholm County Council. Both the individually-tailored and disorder-specific ICBT were 12-week courses with asynchronous written therapist-guidance given by licensed psychologists or psychology students under supervision. Post treatment follow-up were at the same time point at 12 weeks after treatment start for all treatments. All treatments also used similar content based on CBT self-help texts and very similar routines in case of emergencies like participant suicidality. Recruitment to TAIL was done at primary care units within the context of a randomised controlled trial (the REGASSA study) where media recruitment was used in combination with recruitment of patients in regular care, while the participants of DS treatments were mostly self-referred.

The patients included in the REGASSA study were randomised to either TAIL, monitored physical exercise, or treatment as usual, while the patients in DS in the vast majority of cases received the interventions they initially applied for. In TAIL, to be classified to have a probable diagnosis of panic disorder or social anxiety disorder, the participant had to self-report recognition and severity of the condition in question during the initial tailoring-period of treatment, while in DS panic and DS social anxiety, the diagnoses were assessed by a psychiatrist using the M.I.N.I. semi-structured interview. Supplementary Table 1 reports demographics and use of treatments.

It is also important to note that albeit slight differences in recruitment and screening, both groups demonstrated similar symptom pre-treatment scores on each of the outcomes variables; with no significant between groups differences observed upon testing at baseline (MADRS-S _Group|baseline_ Wχ² = 1.08, *p* = .30; LSAS _Group|baseline_ Wχ² = 0.17, *p* = .68; PDSS-R _Group|baseline_ Wχ² = 1.18, *p* = .28). This result suggests clinical similarity between the two populations. In addition, patient satisfaction was good in both TAIL and DS treatments. The Client Satisfaction Questionnaire-8 item version (CSQ-8) ^16^ measures satisfaction with treatment with scores from 8 to 32. Cut-offs described by Smith and colleagues ^17^ states that a CSQ-8 score of 8–13 indicates poor, 14–19 fair, 20–25 good and 26–32 excellent satisfaction. The mean (sd) CSQ-8 score of TAIL was 23.66 (6.12) compared to 24.51 (4.75) for DS depression. ^3^

**Study flow**

In all groups, participants with minimal symptoms at baseline (MADRS-S <13; PDSS-SR <6; LSAS-SR <30 respectively) were excluded from analysis in this study since the Internet psychiatry clinic to some extent includes patients with a diagnosis but low initial severity and thus little room for symptom improvement, which was not the case in the REGASSA study. In TAIL, 43 cases of missing MADRS-S data before treatment, and 64 cases after treatment, were imputed from clinician-rated MADRS at the same time point via regression imputation. ^18^ See supplementary Fig. 1 for a flow chart of this benchmarking study.

**Sensitivity analysis**

To ensure that the statistical testing of relative group effectiveness was unbiased, additional statistical tests were conducted with the aims of identifying confounding influences or interference from demographical differences between groups. To determine evidence of confounding or interference, a sensitivity analysis was conducted with demographical information such as marital status, employment status and gender used as adjustment covariates alongside the main analysis of the group by time model (e.g. a model with group, time, group by time, marital status, employment status, gender). In this way, any influence associated with demographical differences between the samples can be quantified and adjusted for.

The inclusion of marital status and employment status were significantly associated with the outcome in panic treatment (Wχ²_relationship status_ = 8.18, *p* < .01, exp(*β*)=1.10; Wχ²_employment status_ = 3.97, *p* = .046, exp(*β*)=0.86), depression treatment (Wχ²_relationship status_ = 12.32, *p* < .01, exp(*β*)=1.06; Wχ²_employment status_ = 7.52, *p* < .01, exp(*β*)=0.93) but not social anxiety treatment (Wχ²_relationship status_ = 2.96, *p* = .09, exp(*β*)=1.13; Wχ²_employment status_ = 0.14, *p* = .71, exp(*β*)=0.86). No gender differences were identified in any treatment. Next, the estimates of change within each group, and the test of a group by time interaction, were examined within the unadjusted and adjusted models. These adjustments were repeated in the modeling of each treatment, with the results presented in supplementary Table 2, under the row heading of adjusted and unadjusted models.

Together, although marital status and employment were associated with both depressive and panic symptoms, the inclusion of these covariates as adjustment variables did not modify any of the six estimates of the marginal means, or the interpretation of the statistical testing of the time by group interaction.

**Supplementary Fig. 1** Flow chart of benchmarking study. DS, Disorder-specific treatment; TAIL, Individually-tailored treatment.

Excluded

LSAS-SR <30 (n=140)
No social anxiety (n=44)

Excluded

PDSS-SR <6 (n=204)
No panic (n=59)

Excluded

MADRS-S <13 (n=33)

Excluded

LSAS-SR <30
(n=46)

Excluded

PDSS-SR <6
(n=166)

Excluded

MADRS-S <13
(n=155)

DS Internet psychiatry clinic

TAIL within the REGASSA RCT (n=317)

TAIL panic

TAIL

depression

(n=2513)

TAIL social anxiety

## Baseline (Pre)

## Follow-up (Post)

Included

(n=54)

Included

(n=46)

Included

(n=284)

Included

(n=1335)

Included

(n=2358)

Included

(n=1176)

Answered post

(n=953, 81%)

Analysed

(n=1176)

Answered post

(n=1096, 82%)

Analysed

(n=1335)

Answered post

(n=1980, 84%)

Analysed

(n=2358)

Answered post

(n=39, 72%)

Analysed

(n=54)

Answered post

(n=34, 74%)

Analysed

(n=46)

Answered post

(n=246, 87%)

Analysed

(n=284)

Missing PDSS-SR
(n=223, 19%)

Missing LSAS-SR
(n=239, 18%)

Missing MADRS-S (n=378, 16%)

Missing PDSS-SR (n=15, 28%)

Missing LSAS-SR (n=12, 26%)

Missing MADRS-S (n=38, 13%)

DS depression

(n=2513)

DS social anx-

iety (n=1381)

DS panic

(n=1342)

**Supplementary Table 1. Demographics and use of treatments**

|  | TAIL depression, n = 284 | DS depression,  n = 2358 | TAIL panic,  n = 54 | DS panic,  n = 1176 | TAIL social anxiety, n = 46 | DS social anxiety, n = 1335 |
| --- | --- | --- | --- | --- | --- | --- |
| **Age** |  |  |  |  |  |  |
| Mean (95% CI) | 43.3 (41.8-44.7)* | 37.4 (36.9-37.9) | 41.5 (38.1-44.8)* | 33.4 (32.8-34.0) | 41.5 (37.5-45.6)* | 32.0 (31.5-32.6) |
| **Gender** |  |  |  |  |  |  |
| Proportion female (95% CI) | .72 (.66-.76) | .66 (.64-.68) | .69 (.55-.79) | .62 (.59-.65) | .67 (.53-.79) | .55 (.53-.58) |
| **Marital status** |  |  |  |  |  |  |
| Proportion married or cohabiting (95% CI) | .48 (.42-.54) | .50 (.48-.53) | .48 (.36-.61) | .60 (.57-.63) | .50 (.36-.64) | .46 (.43-.50) |
| **Employment status** |  |  |  |  |  |  |
| Proportion unemployed (95% CI) | .16 (.12-.20)* | .10 (.09-.12) | .09 (.04-.20) | .07 (.05-.09) | .09 (.03-.20) | .09 (.07-.11) |
| **Number of log-ins** |  |  |  |  |  |  |
| Mean (95% CI) | 44.5 (39.3-49.8) | 40.9 (39.6-42.2) | 52.9 (41.1-64.6)* | 38.4 (36.8-40.0) | 48.7 (38.5-58.9) | 40.8 (39.3-42.3) |
| **Number of sent messages** |  |  |  |  |  |  |
| Mean (95% CI) | 16.6 (14.8-18.3)* | 14.7 (14.3-15.0) | 18.7 (14.6-22.7)* | 15.3 (14.8-15.8) | 17.6 (14.0-21.2)* | 14.8 (14.4-15.2) |
| **Number of treatment modules** |  |  |  |  |  |  |
| Mean (95% CI) | 6.99 (6.39-7.59) | 7.18 (7.06-7.30) | 8.07 (6.72-9.43)* | 7.10 (6.93-7.26) | 8.24 (6.76-9.72) | 7.66 (7.48-7.85) |
| **Total therapist-time, minutes** |  |  |  |  |  |  |
| Mean (95% CI) | 175 (156-194) | 174 (169-179) | 200 (156-245)* | 144 (139-150) | 207 (160-253)* | 163 (157-168) |

TAIL, Individually-tailored treatment; DS, Disorder-specific treatment; * = Statistically significant difference between TAIL and DS group, *p* < .05.

**Supplementary Table 2. Sensitivity analysis with models adjusted for potential confounding variables.**

|  | Estimated marginal means | | | | Percentage change |
| --- | --- | --- | --- | --- | --- |
|  | n | Pre (s.e.) | Post (s.e.) | G x T sig. | Percentage change (95% CI) |
| **Depression (MADRS-S)** |  |  |  |  |  |
| DS depression *unadjusted* | 2358 | 23.57 (.12) | 13.81 (.19) | W*χ²* = 0.00 | 41.3% (39.7-42.8) |
| DS depression *adjusted* |  | 23.94 (.28) | 14.21 (.27) | W*χ²* = 0.06 | 40.6% (38.3-42.7) |
|  |  |  |  |  |  |
| TAIL depression *unadjusted* | 284 | 22.98 (.35) | 13.50 (.52) | *p* = .98 | 41.3% (36.7-45.6) |
| TAIL depression *adjusted* |  | 23.52 (.43) | 13.83 (.55) | *p* = .81 | 41.3% (36.4-45.5) |
| **Panic (PDSS-SR)** |  |  |  |  |  |
| DS panic *unadjusted* | 1176 | 12.30 (.13) | 5.56 (.15) | W*χ²* = 3.52 | 54.5% (52.0-56.9) |
| DS panic *adjusted* |  | 13.00 (.44) | 5.86 (.27) | W*χ²* = 3.97 | 55.7% (51.2-59.3) |
|  |  |  |  |  |  |
| TAIL panic *unadjusted* | 54 | 12.02 (.60) | 6.67 (.81) | *p* = .06 | 43.9% (29.0-55.6) |
| TAIL panic *adjusted* |  | 12.77 (.77) | 7.08 (.86) | *p* = .051 | 44.5% (29.9-56.4) |
| **Social anx. (LSAS-SR)** |  |  |  |  |  |
| DS social anxiety *unadjusted* | 1335 | 73.15 (.62) | 53.79 (.74) | W*χ²* = 0.22 | 26.5% (24.5-28.5) |
| DS social anxiety *adjusted* |  | 78.00 (1.32) | 55.37 (1.66) | W*χ²* = 0.94 | 29.0% (24.7-33.1) |
|  |  |  |  |  |  |
| TAIL social anxiety *unadjusted* | 46 | 70.50 (3.39) | 53.45 (3.82) | *p* = .64 | 24.2% (12.8-34.1) |
| TAIL social anxiety *adjusted* |  | 75.55 (3.74) | 57.21 (4.33) | *p = .33* | 24.2% (12.1-34.7) |

Comparison of disorder-specific and individually-tailored treatments, unadjusted and adjusted models. TAIL, Individually-tailored treatment; DS, disorder-specific treatment; W*χ²*, Wald Chi-square; G x T, Interaction effect of group and time from generalized estimating equations; MADRS-S, Montgomery-Åsberg Depression Rating Scale - Self rated; PDSS-SR, Panic Disorder Severity Scale - Self report; LSAS-SR, Liebowitz Social Anxiety Scale - Self-Rated.

**Additional references**

16 Attkisson CC, Zwick R. The client satisfaction questionnaire. Psychometric properties and correlations with service utilization and psychotherapy outcome. *Eval Program Plann* 1982; **5**: 233–7.

17 Smith D, Roche E, O’Loughlin K, Brennan D, Madigan K, Lyne J, *et al.* Satisfaction with services following voluntary and involuntary admission. *J Ment Health Abingdon Engl* 2014; **23**: 38–45.

18 Hedman E, Ljótsson B, Blom K, El Alaoui S, Kraepelien M, Rück C, *et al.* Telephone versus internet administration of self-report measures of social anxiety, depressive symptoms, and insomnia: psychometric evaluation of a method to reduce the impact of missing data. *J Med Internet Res* 2013; **15**: e229.
